# The investigation of relationship between serum melatonin levels with Beck Depression Inventory and Beck Scale for Suicidal Ideation in suicide patients

**DOI:** 10.1590/1806-9282.20231614

**Published:** 2024-07-19

**Authors:** Aynur Yurtseven, Cemil Kavalci, Yasemin Yilmaz Aydin, Kemal Aydin, Ömer Faruk Demir, Şeyda Özdemir, Gülsüm Kavalci

**Affiliations:** 1 Dışkapı Yıldırım Beyazıt Training and Research Hospital, Department of Emergency – Ankara, Turkey.; 2 Health Science of Turkey, Antalya Training and Research Hospital, Department of Emergency – Antalya, Turkey.; 3 ışkapı Yıldırım Beyazıt Training and Research Hospital, Department of Biochemistry – Ankara, Turkey; 4 Antalya Training and Research Hospital, Department of Anesthesiology – Antalya, Turkey.

**Keywords:** Emergency, Depression, Suicide, Melatonin

## Abstract

**OBJECTIVE:**

Melatonin plays a role in many biological and physiological events. There are studies in the literature relating melatonin levels to many psychiatric disorders such as schizophrenia, bipolar disorder, and major depressive disorder. We aimed to investigate the relationship between serum melatonin levels with the Beck Depression Inventory and the Beck Scale for Suicidal Ideation in suicide patients.

**METHODS:**

The study was conducted prospectively with volunteer patients aged 20–50 years who were admitted to the emergency department after a suicide attempt. The social and occupational status, educational levels, marital status, and stressor factors of patients were questioned. Beck Depression Inventory and Beck Scale for Suicidal Ideation were applied to each patient included in the study. Blood melatonin levels were evaluated using the enzyme-linked immunosorbent assay method. The data were analyzed with the SPSS 23.00 statistical program. Descriptive values were expressed by the number of cases (n), percentage (%), median (interquartile range), and mean±standard deviation. The Kolmogorov-Smirnov test was used to assess the distribution of continuous variables, and the Pearson or Spearman correlation test was used to assess the relationship between disease severity and melatonin level. A value of p<0.05 was considered statistically significant.

**RESULTS:**

No statistically significant correlation was found between melatonin level and the Beck Depression Inventory score (r=-0.098, p=0.44). However, a statistically weak, inverse, and significant correlation was discovered between melatonin levels and the Beck Scale for Suicidal Ideation score (r=-0.465, p=0.00).

**CONCLUSION:**

According to our results, it was determined that there was a significant negative relationship between melatonin level and the Beck Scale for Suicidal Ideation scoring.

## INTRODUCTION

Instances of suicide exhibit a wide range of clinical manifestations, which -can vary significantly, and in severe situations, may lead to fatality^1^. Based on data provided by the World Health Organization (WHO), the annual mortality resulting from suicide attempts exceeds 700,000 individuals, with a yearly escalation rate ranging from 10 to 20 times^2^. Suicide has been shown to account for 8.5% of fatalities in those aged 15–29 years, making it the world's second leading cause of death in this age group^3^. With the continuous growth of the global population, the emergence of suicide cases as a significant health issue has become an unavoidable reality.

The Beck Depression Inventory (BDI) is a psychometric instrument including 21 questions that have been systematically organized to assess various manifestations of depressive symptoms^4^. Numerous research studies have established a correlation between depression and suicide, with a crucial emphasis on the potential influence of emotions of despair, drawing upon the findings of prior research that assert the limited clarity of the BDI in assessing suicide risk.

The BSSI was created by Beck et al. in 1979 specifically for the assessment of individuals who had made suicide attempts^5^. The BSSI is a self-report instrument that utilizes the semi-structured interview format, drawing on the Scale for Suicidal Ideation^5^. The BSSI is a tool consisting of 21 items, especially designed for adult patients with psychiatric symptoms, and aims to evaluate individuals' levels of active suicidal ideation, passive suicide desire, and preparation phases^5^.

Melatonin, which is scientifically referred to as N-acetyl 5-methoxy tryptamine, is a hormone that is produced and released by the pineal gland^6^. Melatonin is a hormone that plays a significant role in several biochemical and physiological processes inside the human body^6^. Melatonin, which is a hormone responsible for regulating the body's circadian rhythm, has been found to possess several therapeutic qualities including sleep regulation, antidepressant effects, anxiety reduction, neuroprotection, anti-inflammatory activity, and analgesic capabilities^7^. Various factors, including light exposure, medication use, endocrine diseases, and hormone levels, might potentially influence the amounts of melatonin in the blood plasma, thus impacting the circadian rhythm. The diurnal variation of melatonin levels in the bloodstream is also seen. The circadian rhythm, which is mostly secreted during nocturnal hours, experiences disruption among those engaged in shift work^8^. Psychotropic medications have the potential to influence the sleep–wake cycle and biorhythms, such as body temperature and hormone levels, by modulating the oscillation between states of sleep and alertness. Several studies have demonstrated the impact of lithium, imipramine, valproate, fluoxetine, selective serotonin reuptake inhibitors (SSRIs), and medications belonging to the opiate category on circadian rhythm^9^. Oral contraceptives have the potential to modify melatonin plasma concentrations through their inhibitory action on CYP1A2^10^. The observation that hypothyroidism results in a notable reduction in plasma melatonin levels implies a potential association between thyroid diseases and the pineal gland^11^.

According to the reports, a decline in melatonin levels is observed in individuals with sleep difficulties, depression^12,13^, and suicide^13-15^. Arioz et al. ^16^ have demonstrated that the administration of melatonin is associated with a reduction in depression-like behaviors. Melatonin is employed as a therapeutic intervention for the management of depression and sleep disturbances, as supported by scientific literature^17-19^. According to the findings of Leone et al. ^20^, it was observed that the administration of melatonin resulted in a reduction in the likelihood of self-harm among individuals in the young age group. The findings presented in this study indicate a potential association between the hormone melatonin and certain psychiatric disorders. The investigation of the association between melatonin levels and suicide remains limited in current research.

The objective of this study was to examine the correlation between serum melatonin levels and the BSSI and BDI scores in individuals who had attempted suicide.

## METHODS

### The design

The study was conducted in a prospective, observational manner after the consent of the ethical committee (decision no. 12/10, dated 31.05.2021) in the Emergency Department of Ankara Diskapi Yildirim Beyazit Training and Research Hospital. The study was conducted in accordance with the Helsinki Declaration. We obtained informed permission forms from the patients who volunteered to participate. The power analysis was conducted before the study using the G*power 3.1.9.4 software tool. The sample size required to achieve 80% statistical power was determined to be 63 patients, assuming a medium effect size and a significance level of p<0.05.

### Participants

The study enrolled volunteer patients who were at least 18 years of age and had been admitted to the emergency department between the hours of 08:00 and 16:00. These patients had a history of using numerous medicines for the goal of self-harm. Blood samples were collected during the initial hour of admission to our department, with meticulous attention given to guaranteeing patient well-being.

The exclusion criteria were as follows:

- People who use drugs or refuse to take part in the study;
- Those having thyroid dysfunction;
- Those working in shifts, and taking melatonin or similar drugs, immunosuppressant drugs, and chemotherapy for cancer diagnosis;
- Those having a sleep problem, taking medical help and antipsychotics, and having had a mental diagnosis in the past;
- Those using oral contraceptive pills;
- Those having Cushing's syndrome and Addison's disease;
- Those who had radiation therapy for the pineal gland earlier.

The participants in the research were surveyed on their social and occupational standing, educational attainment, marital status, reproductive history, and sources of stress. The BDI and the BSSI were administered to each participant enrolled in the research within the emergency department, under the guidance of a psychiatrist. The rationale for concurrently using both measures lies in the literature's observation that the BDI scale does not consistently provide accurate predictive capabilities for suicide attempts.

### Determination of serum melatonin levels

To assess the melatonin level, a total of 5 mL of venous blood samples were obtained from patients upon their arrival. These samples were then carefully put in a standard vacuum tube. Subsequently, the serum samples were subjected to centrifugation at a speed of 1,000 revolutions per minute for a duration of 15 min, all within a time frame of 30 min. Finally, the centrifuged serum samples were kept at a temperature of -70°C to facilitate further analysis. The assessment of melatonin levels was conducted utilizing the enzyme-linked immunosorbent assay (ELISA) technique. Before conducting the test, essential calibration investigations were performed. Liquid chromatography-tandem mass spectrometry (LC-MS/MS) is the gold standard for melatonin measurement due to its high sensitivity and accuracy. In the context of comparing the LC-MS/MS assay and ELISA for the measurement of melatonin levels, the ELISA test was selected due to the observed satisfactory concordance within the lower range (<30 pmol/L). However, it is worth noting that for melatonin levels beyond 30 pmol/L, the ELISA assay yielded considerably lower measurements compared with the LC-MS/MS assay^19^.

### Statistical analysis

The data were analyzed using the IBM SPSS Statistics 23.0 software package. The descriptive data in this study were represented by the number of instances (n) and percentage (%), as well as the median and interquartile range (IQR). The distribution of continuous variables was assessed using the Kolmogorov-Smirnov test. Intergroup comparisons were assessed using the Mann-Whitney U and Kruskal-Wallis tests. The Spearman correlation test was utilized to examine the association between the BDI, BSSI, and the level of blood melatonin. A p<0.05 value was considered statistically significant.

## RESULTS

The research comprised 63 patients who satisfied the criteria. The median age of the patients was 29 years^15^, and 65.1% of the patients were female. The study revealed that there was no statistically significant disparity in melatonin levels across genders (p>0.05) (Table 1). Only 12.7% of the patient population have a higher level of education. The demographic details of the patients are presented in Table 1.

**Table 1:** Melatonin levels based on variables.

Variables		n (%)	Melatonin	p-value
Gender	Male	22 (34.9)	148.38 (946.5)	0.63
	Female	41 (65.1)	203.76 (228.7)	
Chronic disease	Yes	9 (14.3)	122.43 (64.9)	0.03*
	No	54 (85.7)	218.44 (495.20)	
Educational status	No	1 (1.6)	102.55	0.63
	Elementary school	16 (25.4)	210.38 (409.4)	
	Middle school	12 (19)	149.17 (1,508.9)	
	High school	26 (41.3)	217.20 (323.5)	
	University	8 (12.7)	170.84 (236.7)	
Marital status	Single	26 (41.3)	218.44 (393.5)	0.73
	Divorced	6 (9.5)	192.98 (295.9)	
	Married	31 (49.2)	178.92 (418.3)	
Children	Yes	38 (60.3)	179.78 (413.6)	0.34
	No	25 (39.7)	223.18 (515.6)	
Mood	Anxious	9 (14.3)	196.55 (629.3)	0.63
	Depressed	54 (85.7)	210.80 (333.6)	
Stressor factor	Familial	46 (73)	184.73 (385.8)	0.28
	Environmental	17 (27)	243.38 (904.7)	
Thought content	Regular	23 (36.5)	210.38 (279.9)	0.83
	Regret	28 (44.4)	196.85 (698.7)	
	Stressor factor	12 (19)	207.73 (869.7)	
Hospitalization status	Discharged	43 (68.3)	243.38 (708.6)	0.088
	Ward	7 (11.1)	157.18 (1,144.8)	
	ICU	13 (20.6)	149.17 (76.1)	

* Statistical significance.

In the study, it was found that 76.4% (n=50) of participants reported using numerous types of drugs. Additionally, 19.1% (n=12) reported using only one type of drug. A small percentage, i.e., 1.6% (n=1), engaged in jumping from a height following drug use. Furthermore, 3.2% (n=2) reported engaging in suicidal self-mutilation, while another 1.6% (n=1) reported consuming rat poison.

The findings of this study indicate a statistically significant decrease in blood melatonin levels in those with chronic disorders compared with those without chronic diseases (p<0.05). The blood melatonin levels, as influenced by various circumstances, are presented in Table 1.

A significant correlation was found between familial stress variables and suicide in 73% of the individuals under observation. The study revealed that a significant majority of the patients, i.e., 85.7%, had symptoms of depression as seen in their emotional state. Upon analyzing the blood melatonin level in relation to marital status, it was determined that there was no statistically significant difference (p>0.05).

The median BDI score was 25^13^, whereas the median BSSI score was 10^9^. The median value of melatonin level was found to be 197.15 (379.3). A statistically significant correlation was observed between the BDI and the BSSI score, indicating a weak positive relationship (r=0.423, p<0.05).

**Figure 1:**
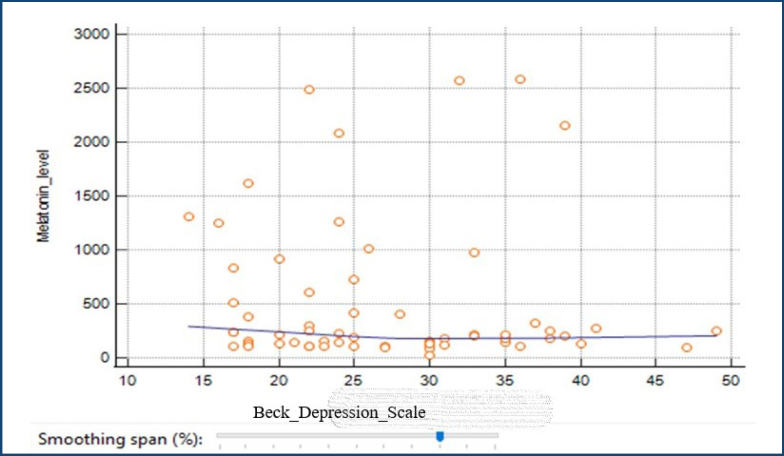
Relationship between Beck Depression Inventory and blood melatonin levels.

**Figure 2:**
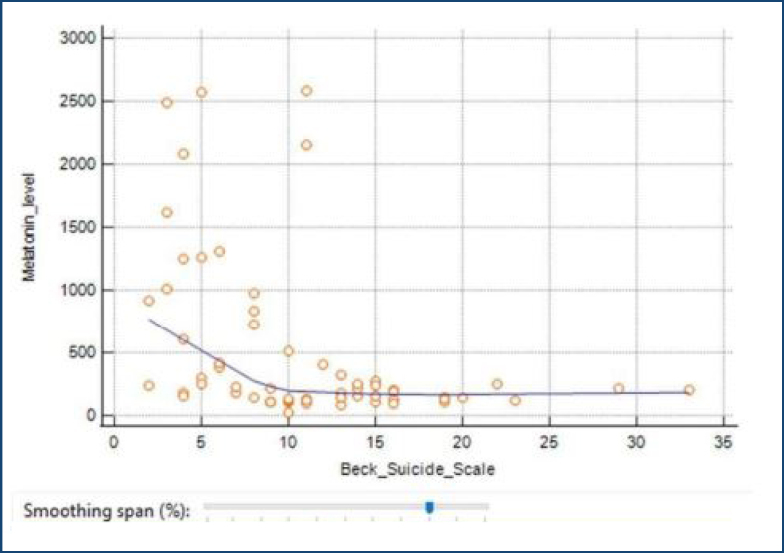
Relationship between Beck Suicide Idea Scale and blood melatonin levels.

The Spearman correlation test was employed to ascertain the presence of an association between melatonin levels and the BDI as well as the BSSI. The results of the study indicate that there is no statistically significant relationship between melatonin levels and BDI scores (r=-0.098, p>0.05) (see Figure 1). Nevertheless, an analysis of the data revealed a statistically significant, moderate, and inverse association between melatonin levels and the BSSI (r=-0.511, p<0.05) (see Figure 2). It was observed that there was a positive correlation between the BSSI and the reduction in melatonin levels.

## DISCUSSION

Melatonin (N-acetyl-5-methoxytryptamine), which has both hydrophilic and lipophilic affinities, is produced by the pineal gland and easily diffuses into membranes, cytoplasm, nucleus, and mitochondria and can be used safely even at high doses^21,22^. Melatonin is involved in many physiological events, from the regulation of the sleep–wake cycle and circadian rhythms in the body, neural development, jet lag treatment, and improvement of the cardiovascular system to the regulation of the immune system and endocrine functions^23,24^. Melatonin is effective in the synthesis and release of hypothalamic gonadotropin-releasing hormone (GnRH) and is involved in the regulation of gonadal physiological responses^25^. Evidence for sex differences in melatonin levels has been reported in many previous studies and has been exhibited in women, suggesting that hormonal contraceptive pills may be a reason for the significant variability in women. Indeed, there is evidence that the use of oral contraceptive pills increases melatonin levels, although many studies do not consider female hormonal status^26^. In this study, in which OCS use was stopped in line with the information in the literature, it was observed that there was no statistically significant difference in melatonin levels between genders (p>0.05).

The findings collected from the study indicate that there is no statistically significant association between the outcomes of the BDI and blood melatonin levels. However, when the relationship between melatonin concentration in the blood and the BSSI score was examined, it was determined that there was a negative correlation, and among individuals with suicidal behavior, those with high blood melatonin levels received lower scores on the BSSI (r=-0.511, p<0.05).

Previous research has suggested that melatonin therapy is particularly effective in addressing sleep disturbances associated with psychiatric conditions, including anxiety and depression, and may even mitigate the risk of suicide^27,28^. Rao et al. ^12^ have shown a reduction in melatonin levels among those with depression and those who have attempted suicide. According to Leone et al. ^29^, the likelihood of suicide attempts in early adolescence was shown to be highest prior to melatonin medication but decreased following the treatment. In their study, Hϕier et al. examined the association between the use of melatonin and suicidal behavior. The results indicated that those who were prescribed melatonin exhibited a suicide rate that was four times higher and a risk of initial suicide attempt that was five times greater in comparison with those who did not get melatonin prescription^30^. The study encompassed a substantial population sample in Denmark, yielding valuable insights into various psychiatric and sleep disorders linked to suicidal behaviors. However, the fact that almost all of the individuals who exhibited suicidal behavior and received melatonin treatment also had a history of an accompanying mental disorder was seen as a limitation of the study. This study was designed by reviewing previous studies and current literature information, and many factors that could affect blood melatonin levels were excluded from the study.

In this investigation, it was shown that a negative correlation existed between the concentration of melatonin and the BSSI score. There is a positive correlation between the reduction in melatonin levels and the increase in suicidal thoughts. Reduced levels of melatonin have been associated with sleep disruptions and the potential to elicit suicidal ideation.

## CONCLUSION

According to our findings, there is an inverse relationship between the melatonin level and BSSI. As the melatonin level decreases, the BSSI score in creases. In line with the information obtained from this study, it is recommended to start exogenous melatonin treatment before an active suicidal attempt occurs in individuals who exhibit suicidal thoughts and decrease endogenous melatonin levels. We think that at least some of the suicide attempts can be prevented with exogenous melatonin treatment.

### Limitations of the study

In this investigation, individuals with comorbid neuropsychiatric conditions undergoing treatment, engaging in shift work, experiencing sleep disturbances, or using drugs known to potentially induce sleep disturbances were excluded from participation. This study aimed to address the inadequacies and limitations identified in prior research, thus making a valuable contribution to the existing body of knowledge due to its accessibility.

## INFORMED CONSENT

Written informed consent was obtained from all subjects before the study.

## ETHICAL APPROVAL

Ethical approval for this study was obtained from Dişkapi Yildirim Beyazıt Training and Research Hospital (31.05.2021, 112/10). This study was performed according to the Declaration of Helsinki.
